# Mind your assays: Misleading cytotoxicity with the WST-1 assay in the presence of manganese

**DOI:** 10.1371/journal.pone.0231634

**Published:** 2020-04-16

**Authors:** Eleonora Scarcello, Alexia Lambremont, Rita Vanbever, Pascal J. Jacques, Dominique Lison

**Affiliations:** 1 Louvain centre for Toxicology and Applied Pharmacology, Université catholique de Louvain, Brussels, Belgium; 2 Institute of Mechanics, Materials and Civil Engineering, Université catholique de Louvain, Louvain-la-Neuve, Belgium; 3 Advanced Drug Delivery & Biomaterials, Louvain Drug Research Institute, Université catholique de Louvain, Brussels, Belgium; West Virginia University School of Medicine, UNITED STATES

## Abstract

The WST-1 assay is the most common test to assess the *in vitro* cytotoxicity of chemicals. Tetrazolium-based assays can, however, be affected by the interference of tested chemicals, including carbon nanotubes or Mg particles. Here, we report a new interference of Mn materials with the WST-1 assay. Endothelial cells exposed to Mn particles (Mn alone or Fe-Mn alloy from 50 to 1600 μg/ml) were severely damaged according to the WST-1 assay, but not the ATP content assay. Subsequent experiments revealed that Mn particles interfere with the reduction of the tetrazolium salt to formazan. Therefore, the WST-1 assay is not suitable to evaluate the *in vitro* cytotoxicity of Mn-containing materials, and luminescence-based assays such as CellTiter-Glo^®^ appear more appropriate.

## 1. Introduction

Since the beginning of the twenty-first century, biodegradable metals represent a new generation of biomedical materials used as temporary implants in vascular intervention or osteosynthesis [1, 2]. These novel biodegradable metals have revolutionized the traditional idea of permanent devices, avoiding persistent physical irritation, chronic inflammation, the need for prolonged anti-platelet aggregation therapy, and possible surgery for removing the implant [3]. Indeed, these materials are designed to corrode *in vivo* while keeping an appropriate therapeutic function until final disappearance. In this context, biodegradable Fe and Mg alloys are promising materials. Corrosion rates for Mg are too fast in both *in vitro* and *in vivo* models [4, 5], Fe emerges as an ideal candidate from preliminary *in vitro* assays [6]. However, animal investigations showed that the pure Fe degradation rate was too slow, as vascular implants remained relatively intact up to a year after implantation [7]. Therefore, novel Fe-based alloys (e.g. Fe-Mn alloys) are developed to expedite the degradation process. The addition of Mn is particularly favorable because it not only enhances the degradation rate but also improves mechanical characteristics [8]. By varying the Mn content and temperature, a deformation mode called Twinning Induced Plasticity (TWIP) can be activated in these alloys, bringing large combinations of strength and ductility [9, 10]. Fe–Mn alloys containing between 20 and 35 wt.% of Mn exhibit remarkable mechanical properties comparable to those of stainless steel 316L alloy [11].

*In vitro* tests are the first assays to high throughput prescreen candidate materials to evaluate their biocompatibility for biomedical applications. The biological evaluation of medical devices defined by the ISO standard 10993–5 requires to perform direct contact as well extracts tests [12]. Tetrazolium salts (e.g., MTT, XTT, WST-1) are the most widely used tools to assess cell metabolic activity [13]. MTT (3-(4,5-dimethyl-2-thiazolyl)-2,5-diphenyl-2H-tetrazolium bromide), XTT (2,3-bis-(2-methoxy-4-nitro-5-sulfophenyl)-2H-tetrazolium-5-carboxanilide) and WST-1 (2-(4-iodophenyl)-3-(4-nitrophenyl)-5-(2,4-disulfophenyl)-2H-tetrazolium) assays rely on the cellular reduction of tetrazolium salts to their formazan products [14]. The WST-1 reagent presents several advantages compared to the two other tetrazolium salt-based cell proliferation reagents, MTT and XTT, including water-solubility, rapidity, greater stability and sensitivity [15]. However, tetrazolium-based assays can be confounded by the presence of reducing chemicals, such as dithiothreitol or mercaptoethanol, L-cysteine or L-ascorbic acid, that supplant mitochondrial dehydrogenases in cleaving tetrazolium salts to a formazan dye [13]. Several papers have highlighted misleading cell viability results when using tetrazolium salts. Wörle-Knirsch *et al*. (2006) [16] demonstrated that single-walled carbon nanotubes bind MTT-formazan crystals suggesting false cytotoxicity results. Monteiro-Riviere *et al*. (2009) [17] showed that carbon black alone could interact with MTT dye and cause false cytotoxicity results. Fischer *et al*. (2010) [18] showed that the corrosion products of Mg-based alloys influence MTT and XTT tests and, more recently, Almutary *et al*. (2016) demonstrated that MTT is a potential confounder in nanoparticle toxicity testing [19].

The impact on surrounding tissues (e.g. endothelium) of high strength Fe-based bioresorbable alloys is considered through the comparison of Twinning Induced Plasticity (TWIP) steel particles and 316L stainless steel particles. The toxicity of these alloys was tested *in vitro* on Human Umbilical Vein Endothelial Cells (HUVEC) lines, selected as the endothelium model. Preliminary cytotoxicity results obtained with two different assays (WST-1 vs. CellTiter-Glo^®^) showed a significant difference. With the CellTiter-Glo^®^ assay the IC_50_ (concentration of TWIP steel particles for which the ATP content was reduced by 50% compared to non-exposed cells after 24h) was >2000 μg/ml, reflecting much lower toxicity than the one measured with the WST-1 assay (IC_50_ of 1000 μg/ml). This difference suggested exploring the phenomenon deeper, considering that interferences and disturbances in viability assays are likely to happen, as described above. Here, we report that the WST-1 assay leads to misleading cytotoxicity results following direct cell exposure to Mn or Mn-containing alloys.

## 2. Materials & methods

### 2.1. Metallic materials

Bars, 15mm in diameter, of Fe-22Mn-0.6C (in wt.%) TWIP steel [9] or 316L stainless steel grade were gas atomized. Induction drip-melting of these bars was used to melt the alloy. The liquid was then atomized with high-pressure Ar. The resulting powder was then sieved in 3 steps. The first one removed particles larger than 120μm. Sieving down to 70μm and then 20μm was then carried out. Carbonyl iron powder (purity > 99.5%) was purchased from Sigma-Aldrich (St Louis, MO) and manganese powder (purity > 99.6%) from Alfa Aesar (Tewksbury, MA). Fe+Mn mixture was obtained by mechanical mixing of 77 wt.% of Fe powder and 22 wt.% of Mn powder. The crystalline silica Min-U-Sil^®^ 5 (Berkeley Springs, WV) was used as positive cytotoxic control.

### 2.2. Material characterization

**Fig 1:**
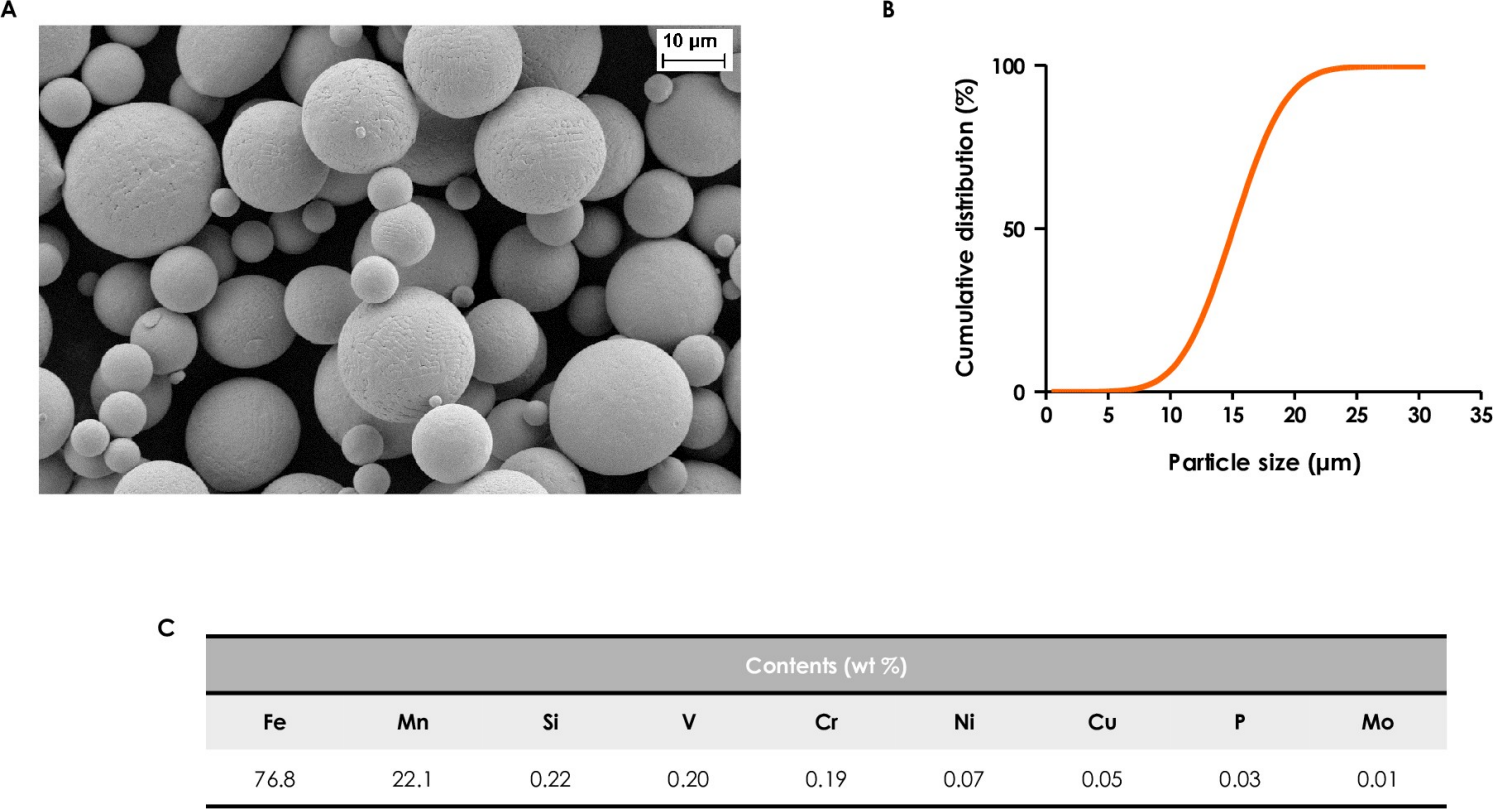
Characterization of TWIP steel powder. (A) SEM micrograph; (B) particle size distribution; (C) elemental composition.

**Fig 2:**
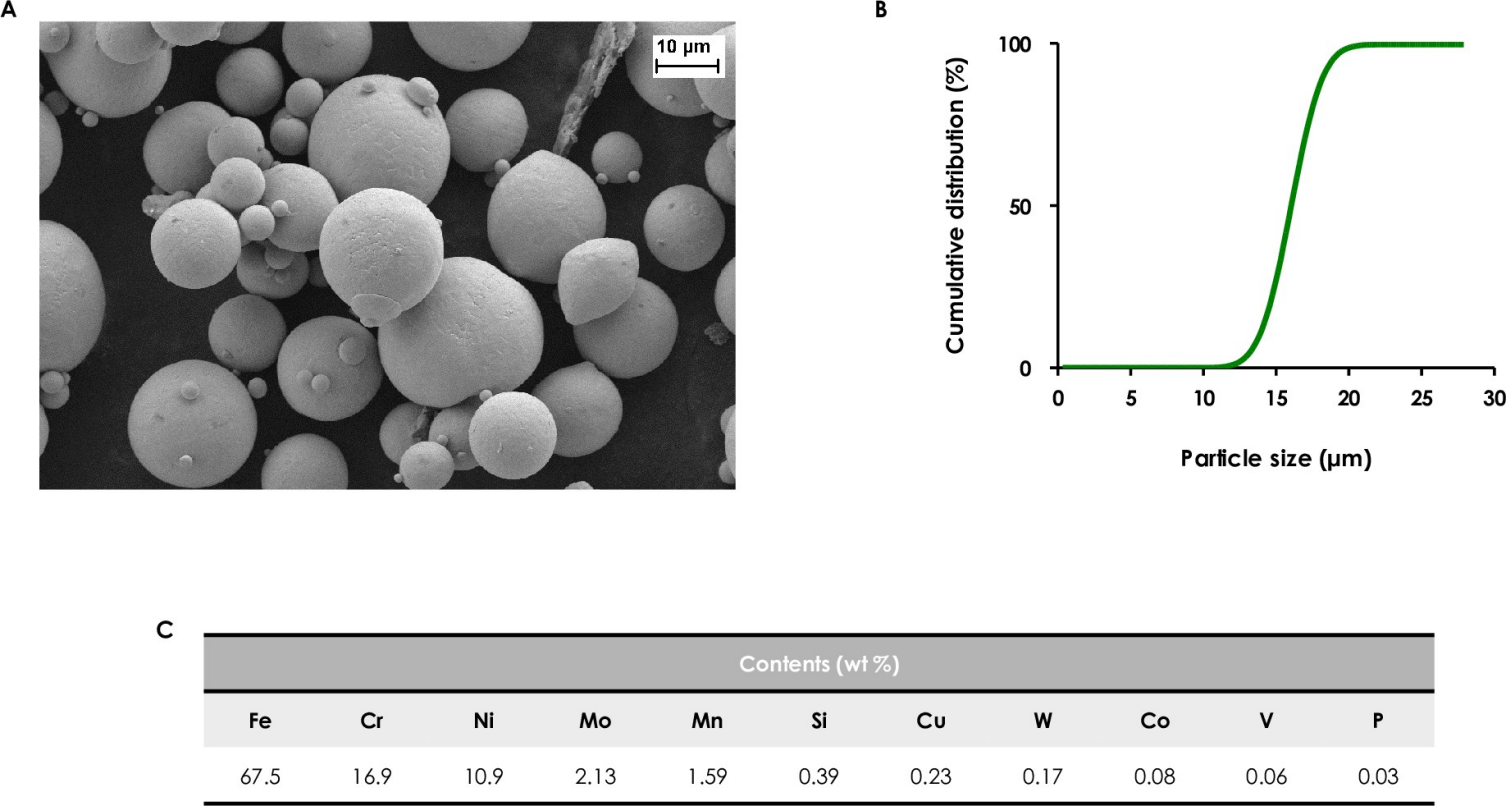
Characterization of 316L steel powder. (A) SEM micrograph; (B) particle size distribution; (C) elemental composition.

The topography and the size of TWIP steel and 316L steel particles was analyzed by scanning electron microscopy (SEM, Ultra55^®^ Zeiss). The cumulative size distribution was obtained by laser diffraction granulometry. The powders were dispersed with compressed air at a pressure of 1.8 bar through a venturi tube (RODOS, Sympatec GmbH, Clausthal-Zellerfelg, Germany) before sizing with a laser diffractometer (HELOS, Sympatec). Increasing concentrations of TWIP steel or 316L steel powders were incubated in endothelial cell growth medium (ECGM) at 37°C for 24h, then suspensions were centrifuged and the chemical concentration of released Fe or Mn ions was quantified by inductively coupled plasma—optical emission spectrometry (ICP-OES, Agilent Technologies 5100) after filtration of the suspension. Characterization of the particles is reported in **Figs 1 and 2** (TWIP and 316L steel powders).

### 2.3. Material sterilization

Immediately before cell exposure particles were heated at 200°C (WTB, Binder^®^ drying oven) during 2h to sterilize the material and inactivate any possible microbial contamination.

### 2.4. Cell culture

Cell studies were performed on Human Umbilical Vein Endothelial Cells (HUVECs, Cell Application, Sigma-Aldrich, San Diego, CA) because they are arguably the best characterized primary human EC type [20]. ECs were cultured on 0.2% gelatin-coated 75-cm^2^ culture flasks (Corning Incorporated, Corning, NY) in Endothelial Cell Growth Medium (Cell Application, Sigma-Aldrich, San Diego, CA), maintained in a humidified incubator (New Brunswick Galaxy^®^ 170S) containing 5% CO_2_ at 37°C. Cells were grown to confluency, harvested by trypsinization and plated in 96-well plates precoated with 0.2% gelatin at a density of 20,000 cells per well. A maximum of 7 passages was used to maintain phenotypic cell characteristics. Cells were routinely tested for the absence of Mycoplasma infection (PCR Mycoplasma Test Kit I/RT, PromoKine, Huissen, Netherlands).

### 2.5. Cell exposure

#### Direct test

After sterilization, the particles were suspended in cell culture medium at a stock concentration of 5 mg/ml, diluted to final concentrations in culture medium and vortexed. Cells were then immediately exposed to different suspensions of particles.

#### Extracts test

Cells were exposed to corrosion extracts generated after immersion of increasing concentrations of particles in cell culture medium for 24h and centrifugation at 1000 rpm/25°C/5’ following ISO standard 10993–5. The concentration of soluble Fe or Mn ions in the supernatant was quantified by ICP-OES (Agilent Technologies 5100) after filtration of these suspensions (**S1 Fig**). Sample were attacked with an acid solution (1:1 HCl and HNO_3_) before ICP-OES analysis (axial reading). Wavelengths used were as follows: 396.847 nm (Ca), 238.204 nm (Fe), 766.491 nm (K), 279.553 nm (Mg), 588.995 nm (Na), 213.618 nm (P) and 181.972 nm (S).

### 2.6. Cell viability assays

#### WST-1 test

The colorimetric WST-1 kit was purchased from Roche Diagnostics GmbH (Mannheim, Germany). In the WST-1 assay, the amount of formazan dye formed is directly related to the metabolic activity of cells. The assay was carried out as described in the manufacturer’s instructions. In brief, cells were seeded in transparent 96-well plates and exposed immediately or the day after to different concentrations of particles or equivalent extracts for 0 or 24h. Cells were then washed twice with DPBS 1X and incubated in fresh medium with 10% WST-1 reagent for 2h. Absorbance was measured at 450 nm (690 nm was used as reference wavelength and subtracted) in a multiplate reader (Infinite F200, Tecan^®^). For some experiments, the full visible spectrum was recorded. Results are reported as relative WST-1 activity, where 1.0 corresponds to the absorbance measured in control cultures. Optical microscopy images of cells were taken with ZEISS Axiocam MRc microscope camera.

#### ATP test

The luminescent CellTiter-Glo^®^ 2.0 assay was obtained from Promega Corp. (Madison, WI). This assay quantifies the amount of ATP in metabolically active cells. In brief, cells were seeded in white 96-well plates in ECGM and exposed and washed as above. The CellTiter-Glo^®^ reagent was added and luminescence read on a luminometer (Victor^™^ X4, PerkinElmer^®^). Results are reported as for WST-1.

An outline of materials and performed cytotoxicity tests is reported in **Table 1**.

**Table 1:** Materials used, test type, and cell viability assays.

	Exposure	Assay	Time (h)
**TWIP steel particles**	Direct Extracts	WST-1, ATP WST-1, ATP	0, 24 0, 24
**316L steel particles**	Direct Extracts	WST-1, ATP WST-1, ATP	0, 24 0, 24
**Fe particles**	Direct	WST-1	0
**Mn particles**	Direct	WST-1	0
Fe+Mn* **particles**	Direct	WST-1	0

*Fe+Mn: mixture of 77 wt.% of Fe powder with 22 wt.% of Mn powder.

### 2.7. Acellular assays

In order to investigate the possible mechanism of interaction between particles and cells, different experiments were performed:

- We determined if the light absorbance is influenced by the presence of TWIP steel particles. Increasing concentrations of TWIP steel particles were thus suspended in the ECGM and added in a transparent 96-well plate without cells. Fresh medium with 10% of WST- reagent was added to the suspension.
- We next tested if TWIP steel particles quench the absorbance of the formazan product. The formazan dye was thus produced by metabolically active cells (HUVECs) or by L-Ascorbic acid (0.5 mM, Fluka, San Diego, CA) from water-soluble tetrazolium salt (WST-1) and added to increasing concentrations of TWIP steel or 316L steel particles in a transparent 96-well plate without cells.
- We explored if TWIP steel particles react with the WST-1 reagent and block the formazan formation. Increasing concentrations of TWIP steel or 316L steel powders were thus incubated with 10% WST-1 reagent in a transparent 96-well plate without cells. After 1 min, the L-Ascorbic acid (0.5 mM) was added to the plate.

After each of these assays, the absorbance was read at 450 nm and connected for absorbance at 690 nm.

### 2.8. Statistical analysis

Values are presented as means ± standard error of the mean (S.E.M.) of experiments conducted in replicates (n). Data were analyzed with GraphPad Prism 5.0 (GraphPad Software, La Jolla, CA) and/or Microsoft Excel. Differences between control and treated groups were analyzed by one-way analysis of variance (ANOVA) followed by a post-hoc Dunnett’s pairwise comparison or a linear trend test to document a dose-dependent effect. Differences with *p* value < 0.05 were considered statistically significant.

## 3. Results

### 3.1. Interference between TWIP steel particles and WST-1 assay

**Fig 3:**
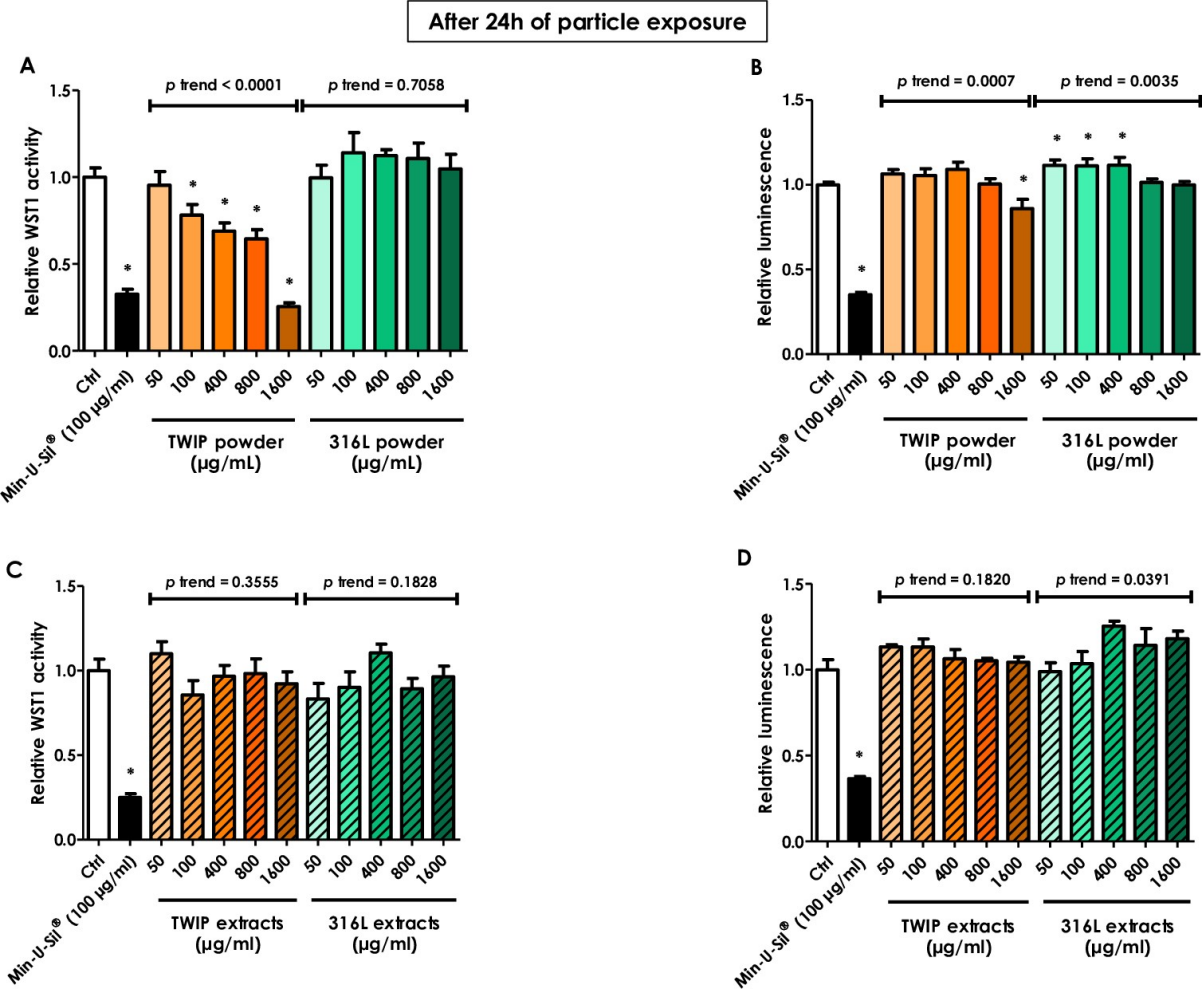
HUVECs WST-1 activity is dose-dependently reduced by direct exposure to TWIP steel particles. HUVECs were grown in 96-well transparent or white plates and exposed the next day to different concentrations of TWIP steel or 316L steel particles or extracts for 24h. Extracts were obtained from culture medium incubated during 24h with the same concentrations of particles. The cells were then washed and further incubated in fresh medium with 10% WST-1 reagent for 2h. Absorbance was measured at 450 nm, with 690 nm as reference (A, C). After 24h exposure and wash, the white plate was replenished with fresh medium containing the CellTiter-Glo^®^ reagent (ATP) and luminescence was read (B, D). Results are reported as relative WST-1 activity or luminescence, where 1.0 corresponds to the value measured in control cultures. Min-U-Sil^®^ was used as a positive control. Values are means ± SEM (n = 8), *p < 0.05 relative to control (One-way ANOVA followed by a Dunnett’s multiple comparison). The trend test included controls.

The HUVECs response to TWIP steel or 316L steel particles or corrosion extracts was examined using two different assays based on different readouts, mitochondrial dehydrogenases (colorimetry) and ATP content (luminescence). Min-U-Sil^®^ was used as positive cytotoxic control. Whilst TWIP steel corrosion extracts did not affect cell viability in both assays, the direct contact with the particles showed an apparent dose-dependent reduction of cell viability in the WST-1 test, less pronounced in the luminescence assay (**Fig 3**. Pictures of cells after particle exposure are shown in **S2 Fig**). The response to the positive control (Min-U-Sil^®^) was similar in both assays. Direct or extracts exposure to the stainless steel 316L particles did not affect ECs viability, except a minor dose-dependent reduction of the luminescence signal which was mainly due to a slight increase of the ATP content at low concentrations (50, 100 and 400 μg/ml) (Panel B).

The quantification of Fe or Mn ions released from TWIP powder reveals a dose-dependent increase, whereas it remains unchanged for 316L steel powders (**S1 Fig**).

**Fig 4:**
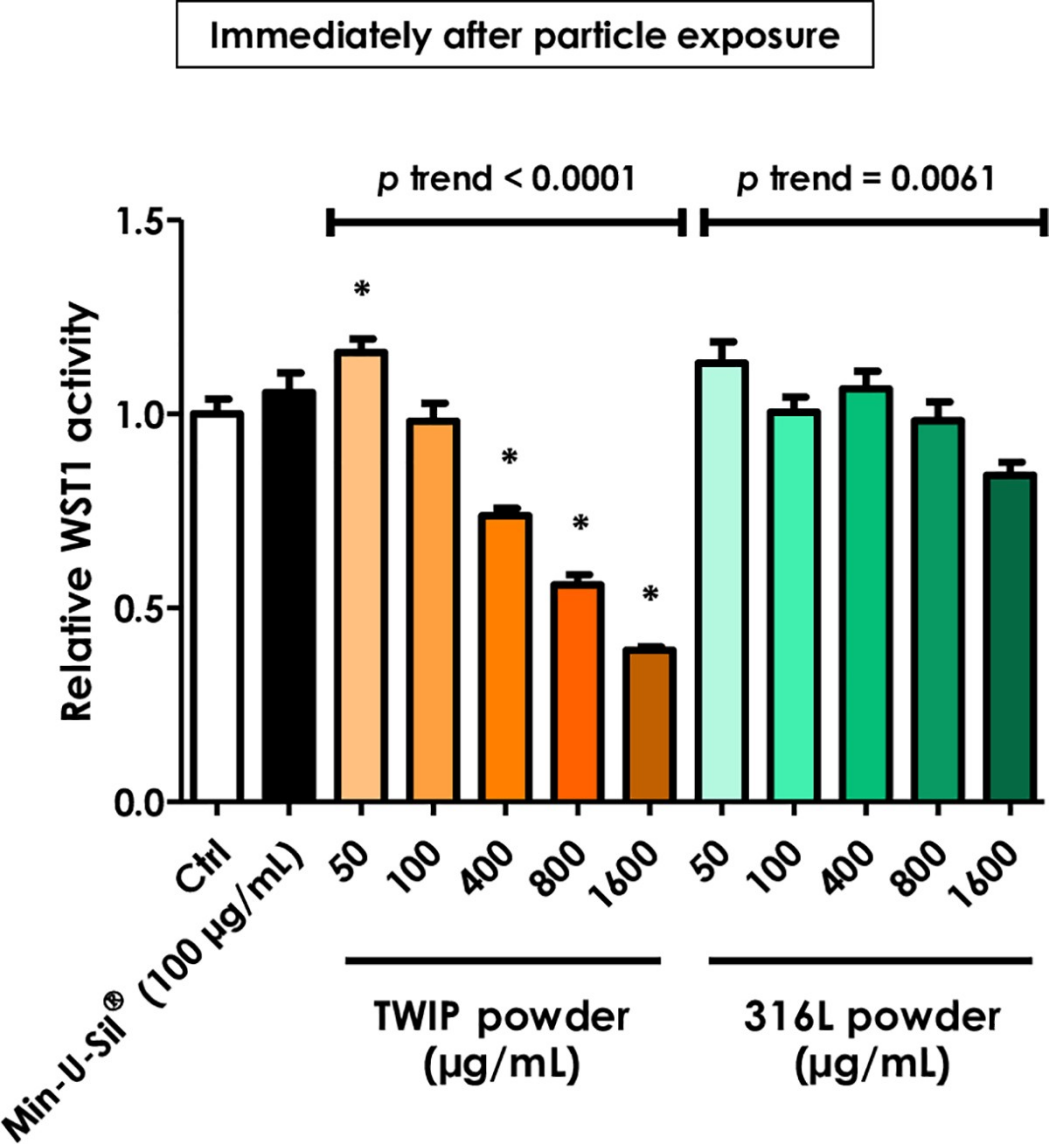
The WST-1 assay falsely indicates that HUVECs are dose-dependently affected by direct exposure to TWIP steel particles. HUVECs were grown in 96-well transparent or white plates and exposed the next day to different suspensions of TWIP steel or 316L steel powders. WST-1 activity was measured immediately after exposure without washing and recorded as shown in Fig 3. Min-U-Sil^®^ was used as positive control. Values are means ± SEM (n = 4), *p < 0.05 relative to control (One-way ANOVA followed by a Dunnett’s multiple comparison). The trend test included controls.

In order to investigate why, after direct exposure, the WST-1 assay indicated significant dose-dependent cytotoxicity compared to the luminescence test, we added the WST-1 reagent immediately after addition of the TWIP steel particles to avoid the longer contribution of cytotoxicity. We measured the colorimetric signal after 2h of incubation and the WST-1 assay showed a reduction of formazan formation in the presence of TWIP steel powder. This reduction did not appear with 316L steel particles (**Fig 4**). Cells exposed to Min-U-Sil^®^ did not show reduced WST-1 activity confirming that the protocol did not allow particles to exert a cytotoxic activity.

### 3.2. Manganese causes the WST-1 interference

**Fig 5:**
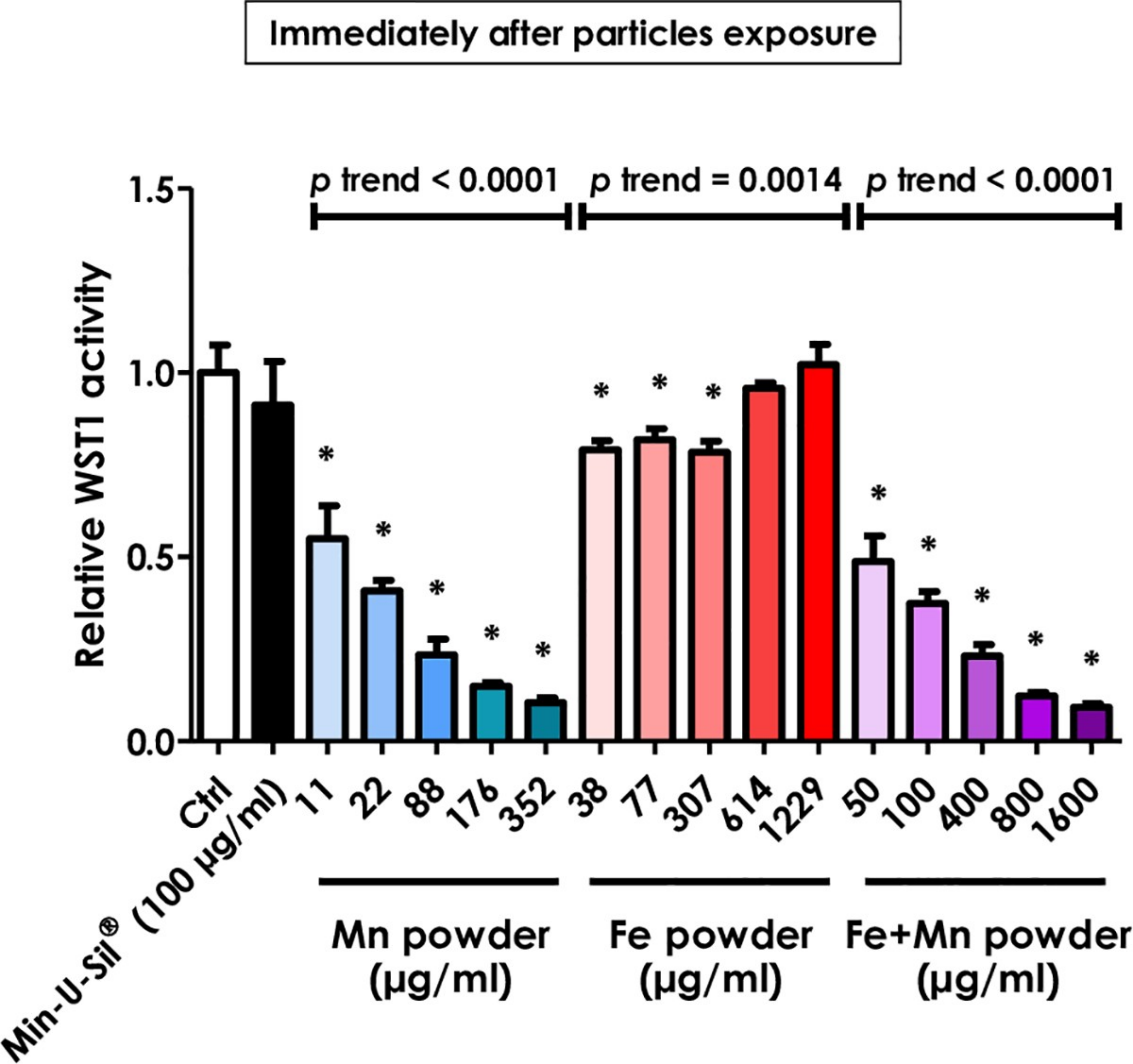
The WST-1 assay indicates a false cytotoxicity after direct addition of Mn powder to HUVECs. HUVECs were seeded in 96-well plates and exposed the next day to different concentrations of Mn, Fe, or Fe+Mn powder. The cells were not washed and immediately incubated in fresh medium with 10% WST-1 reagent for 2h. Absorbance was measured at 450 nm, with 690 nm as reference. Results are reported as relative WST-1 activity, where 1.0 corresponds to the value measured in control cultures. Min-U-Sil^®^ was used as positive control. Fe+Mn: mixture of 77 wt.% of Fe powder with 22 wt.% of Mn powder. Values are means ± SEM (n = 8), *p < 0.05 relative to control (One-way ANOVA followed by a Dunnett’s multiple comparison). The trend test included controls.

Since TWIP steel is an alloy mainly composed of Fe and Mn, we then wanted to discern the potential interference of each element by testing Fe and Mn individually or mixed (i.e., without forming an alloy). WST-1 activity was measured immediately after addition of the particles to ECs. Mn alone or mixed with Fe particles indicated a false loss in viability, whereas Fe particles alone or Min-U-Sil^®^ showed no decrease of the signal (**Fig 5**). We concluded that Mn caused interference with the WST-1.

### 3.3. TWIP steel particles interfere with the reduction of the tetrazolium salt

To investigate the mechanism of interaction, additional experiments were sequentially performed.

**Fig 6:**
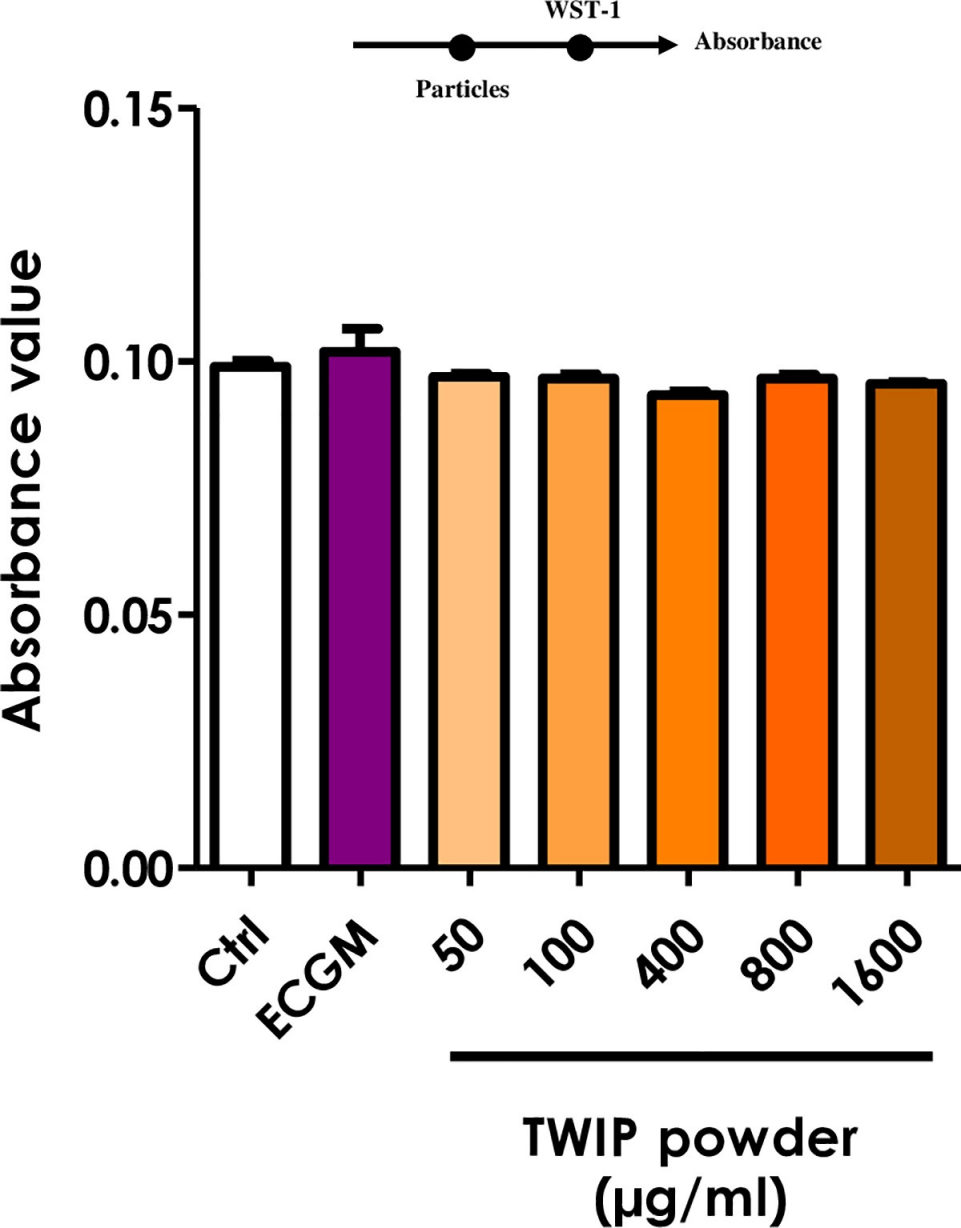
Absorbance is not influenced by TWIP steel particles. Increasing concentrations of TWIP steel powder in ECGM were added into a transparent 96-well plate without cells. Fresh medium with 10% WST-1 reagent was added to the suspensions and the absorbance was read immediately at 450 nm, with 690 nm as reference. Ctrl: cell medium with WST-1 (1:1); ECGM: cell medium without WST-1. Values are means ± SEM (n = 4). On the top: timeline of experiment setup.

**Fig 7:**
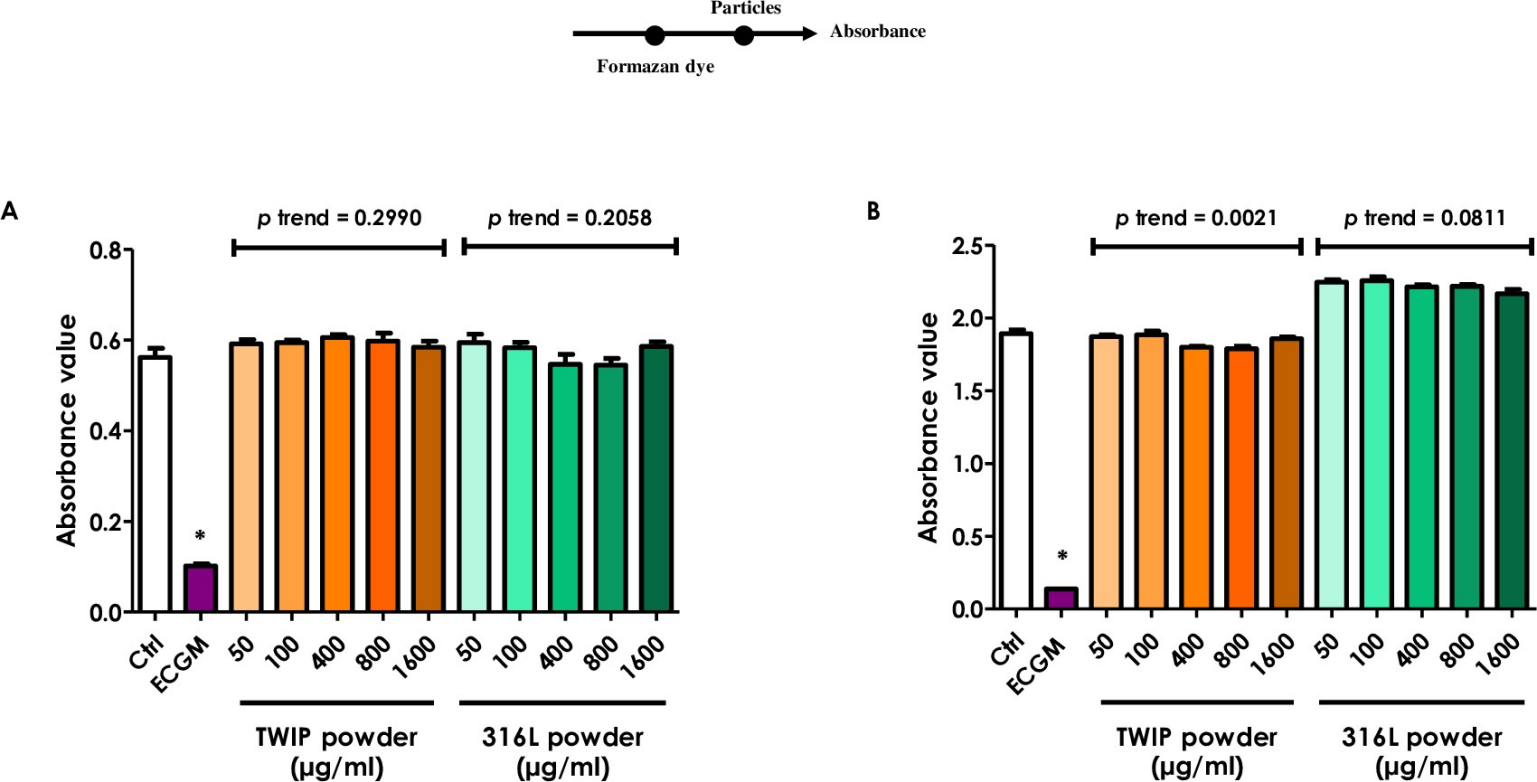
TWIP steel particles do not quench the absorbance of the formazan product. The water-soluble tetrazolium salt (WST-1) was converted by metabolically active cells (HUVECs) or Vitamin C (0.5 mM) into the formazan dye and then added to increasing concentrations of TWIP steel or 316L steel particles. Absorbance was immediately measured at 450 nm, with 690 nm as reference. Results are reported as absorbance value of particles added to the formazan dye cleaved by cells (A), or ascorbic acid (B). ECGM: 100% cell culture medium. Values are means ± SEM (n = 4), *p < 0.05 relative to control (One-way ANOVA followed by a Dunnett’s multiple comparison). The trend test included controls. On the top: timeline of experiment setup.

**Fig 8:**
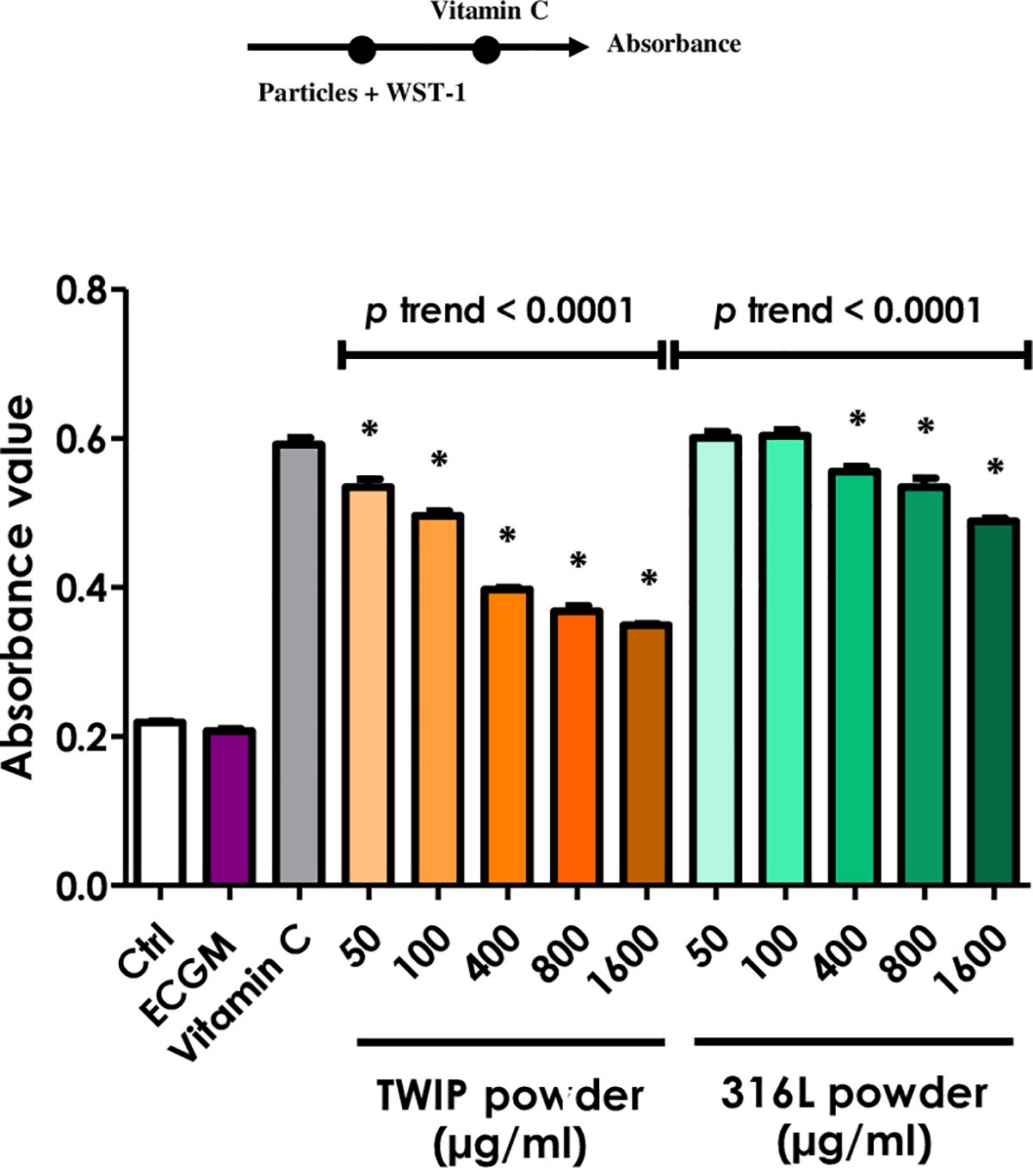
TWIP steel particles react with the WST-1 reagent and block formazan formation. Increasing concentrations of TWIP steel or 316L steel powders were incubated with 10% WST-1 reagent. Then, ascorbic acid (0.5 mM) was added and the absorbance was immediately measured at 450 nm, with 690 nm as reference. Ctrl: 50% ECGM– 50% WST-1; ECGM: 100% cell culture medium; Vitamin C: Vitamin C (0.5 mM) + WST-1. Values are means ± SEM (n = 4), *p < 0.05 relative to control (One-way ANOVA followed by a Dunnett’s multiple comparison). The trend test included controls. On the top: timeline of experiment setup.

When the water-soluble tetrazolium salt was directly added to the particles without cells, the absorbance value at 450 nm was not affected regardless of the powder concentrations (**Fig 6**. This value was obtained after subtraction of the absorbance light at 690 nm, raw data shown in **S3 Fig**). When particles were added to the WST-1-formazan dye obtained from the reduction of WST-1 by cellular mitochondrial dehydrogenases or by ascorbic acid under acellular conditions, no decrease in the absorbance was observed (**Fig 7A and 7B**). Finally, when the reducing agent (ascorbic acid) was added after particles were mixed with WST-1, an evident loss of the absorbance signal was observed with TWIP steel particles, and to a much lesser extent with 316L steel particles (**Fig 8**). Therefore, the Fe-Mn alloy appeared to interfere during the reduction of the WST-1 to formazan, not after the formation of the formazan dye.

**Fig 9:**
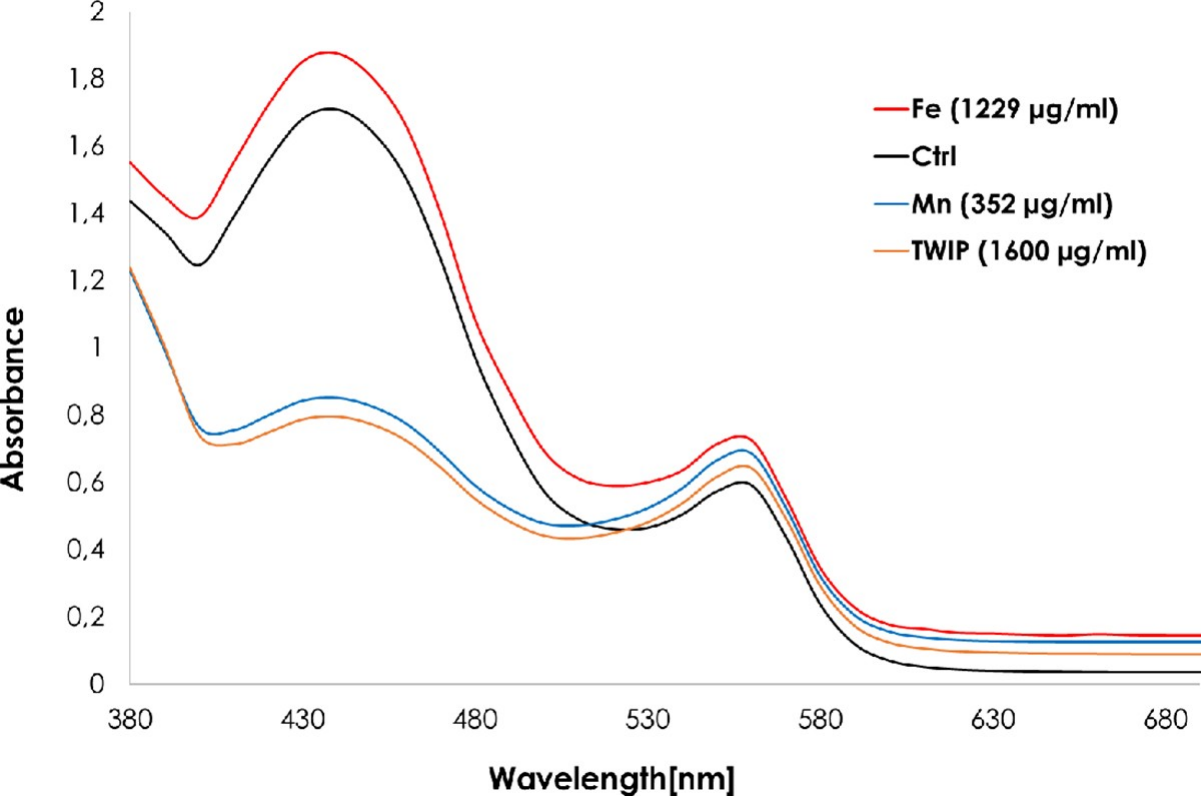
Full visible spectrum of the formazan dye formed in the presence of different particles. Different concentrations of Fe, Mn, TWIP steel or 316L steel powders were incubated with 10% WST-1 reagent. Then, ascorbic acid (0.5 mM) was added and the absorbance was measured at the full visible spectrum.

We speculated that Mn could open the tetrazolium salt ring, bind the nitrogen atom and lead to a change in color different from the formazan generated in the WST-1 test. This new compound would thus produce a spectral shift, observable in the full spectrum recording. We did not record any shift compared to control (**Fig 9**). However, the intensity of the signal at 450 nm was far lower for Mn or TWIP steel particles compared to controls or Fe. Therefore, manganese did not allow the complete formation of formazan (**Fig 9**).

## 4. Discussion

As soon as we talk about biomaterials, questions about their biocompatibility arise. Although biocompatibility involves much more than the absence of toxicity [21], the ISO standard 10993–5 represents the first procedure to follow for determining the possible toxic response of mammalian cells to medical devices [12]. Cell culture is thus fundamental for the initial preclinical screening of potential biomaterials, avoiding expensive *in vivo* experiments. It is, therefore, of vital importance to obtain accurate and reliable results from these *in vitro* cytotoxicity assays [22]. The standard methods to assess *in vitro* cytotoxicity by direct contact or exposure to extracts principally use tetrazolium-based assays because they are sensitive and easy to handle. However, interferences with tetrazolium salts that generate false positive/negative results have been documented since the late 1980s [23]. Several authors have drawn the attention on disturbances of the viability tests leading to misleading results, as in the case of carbon nanotubes considered as “safe” by biomedical researchers but not by toxicologists [24, 25]. Recently, the reliability of the WST-1 results was also challenged in the field of biomaterials, when Mg-based implants revealed false cytotoxic results.

Here, we report that Mn and Mn-containing alloys interact during the conversion of WST-1 into formazan and, as a consequence, indicate a fake loss in cell viability. This strong loss in signal was even observed immediately after the addition of the TWIP steel particles, i.e. in the absence of cytotoxicity. As TWIP steel is a Fe-22Mn-0.6C alloy, each element was tested separately to define which one was responsible for the interference. It appeared that the presence of Mn was crucial for the disturbance of the test. Fe alone did not produce such interference and if both elements–Fe and Mn–were mixed without forming an alloy, the mixture still had the same cytotoxicity effect as Mn alone. Washing the cells before the WST-1 procedure was not able to remove the majority of sedimented metallic particles attached to or internalized by the cells. Remarkably, cells were not affected by metal extracts, indicating that metallic Mn but not ions was involved in the interference.

A further aim of this work was to establish the mechanism of the interaction between manganese and WST-1. We showed that Mn does not interact with formazan dye, formed after reduction of WST-1, but rather while tetrazolium salt is converted into formazan. Indeed, when particles were added to the formed formazan dye, the absorbance value did not change compared to controls (**Fig 7**). However, when TWIP steel particles were present during the conversion of WST-1 to formazan, an obvious loss in the signal was measured (**Fig 8**). 316L steel particles did not produce such an effect, excluding an aspecific particle effect.

One explanation for the interaction during the WST-1 conversion is that Mn is able to open and bind to the ring of WST-1. The new compound causes a change in color similar to the formazan formation, but is detectable at a different wavelength. The full visible absorbance spectrum was thus measured after incubation of particles with WST-1 and the reducing agent (Vitamin C). We did not record any shift of the spectrum compared to control (**Fig 9**). However, the intensity of the signal at 450 nm was far lower for Mn or TWIP steel particles compared to controls or Fe. Therefore, manganese does not allow the complete formation of the formazan (**Fig 9**).

The luminescence-based assay CellTiter-Glo^®^ is a valid alternative to the WST-1 test. Our results did not indicate an interference between Mn particles and the reagent, and slight cytotoxicity was observed only after 24h incubation with the particles.

Overall, we demonstrate here that direct exposure to Mn or Fe-Mn alloys indicates false cytotoxicity results with the WST-1 assay and, therefore, this test is not suitable for the assessment of *in vitro* cell viability with these materials. Luminescence-based assays, e.g. CellTiter-Glo^®^, provide more reliable results and, therefore, are appropriate to evaluate Mn-based materials. At least two or more tests with different readouts should always be performed to evaluate the cytotoxicity of new materials.

## 5. Conclusion

Fe-Mn alloys are considered promising materials for temporary implants such as coronary stents. *In vitro* tests are the first tools to trace a potential for *in vivo* toxicity. Tetrazolium-based assays e.g. WST-1 are widely used to determine the cytotoxicity as they are sensitive, precise, fast and relatively cheap. Here, we show that direct cell exposure to Mn or Fe-Mn alloy, but not Fe or 316L stainless steel particles, leads to apparent misleading cytotoxicity with the WST-1 assay. The same treatment, measured with a luminescence-based assay, did not show such cytotoxicity.

## Supporting information

S1 Fig Quantification of Fe or Mn ions released from TWIP steel or 316L steel powders. Increasing concentrations of (A) TWIP or (B) 316L powder in ECGM medium were incubated at 37°C. After 24h, the suspensions were centrifuged and the chemical concentration of released Fe or Mn ions was quantified by ICP-OES after filtration of the suspension.
(TIF)

S2 Fig Representative optical microscopy images of HUVECs exposed for 24h to 316L steel or TWIP steel powders. HUVECs (20,000 cells/well) were seeded into 96-well plates and exposed the day after to increasing concentration of 316L steel or TWIP steel powders. A 40x magnification image of cells was taken through an optical ZEISS Axiocam microscope camera after 24h of exposure.
(TIF)

S3 Fig Absorbance values of TWIP steel powder at 450 nm (A) and 690 nm (B). HUVECs were cultured on 96-well plates for 24h and then exposed to increasing concentration of TWIP steel powder. After 24h, cells were washed twice with DPBS and incubated in fresh medium with 10% WST-1 reagent for 2h. Absorbance was measured at 450 nm and 690 nm in a multiplate reader (Infinite F200, Tecan^®^).
(TIF)
