# Supplementary figures and images for: Mind your assays: Misleading cytotoxicity with the WST-1 assay in the presence of manganese
Source: PLoS One. 2020 Apr 16;15(4):e0231634. doi: 10.1371/journal.pone.0231634 (PMC7161962; doi:10.1371/journal.pone.0231634)

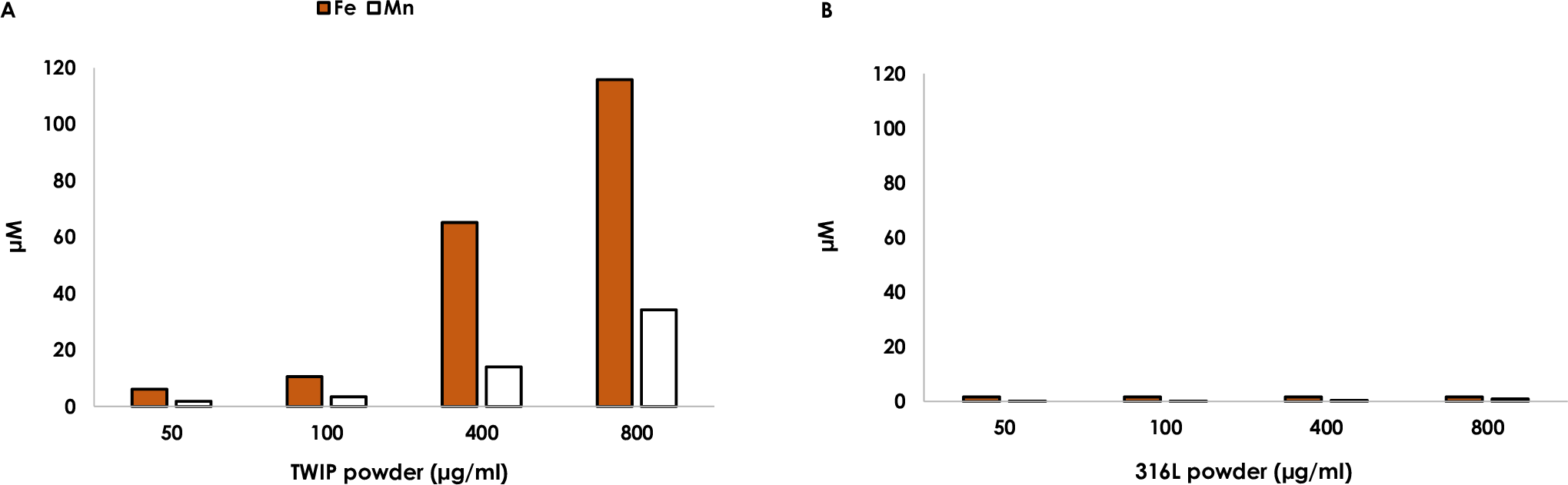

Supplement: S1 Fig — Increasing concentrations of (A) TWIP or (B) 316L powder in ECGM medium were incubated at 37°C. After 24h, the suspensions were centrifuged and the chemical concentration of released Fe or Mn ions was quantified by ICP-OES after filtration of the suspension. (TIF) [file pone.0231634.s001.tif]

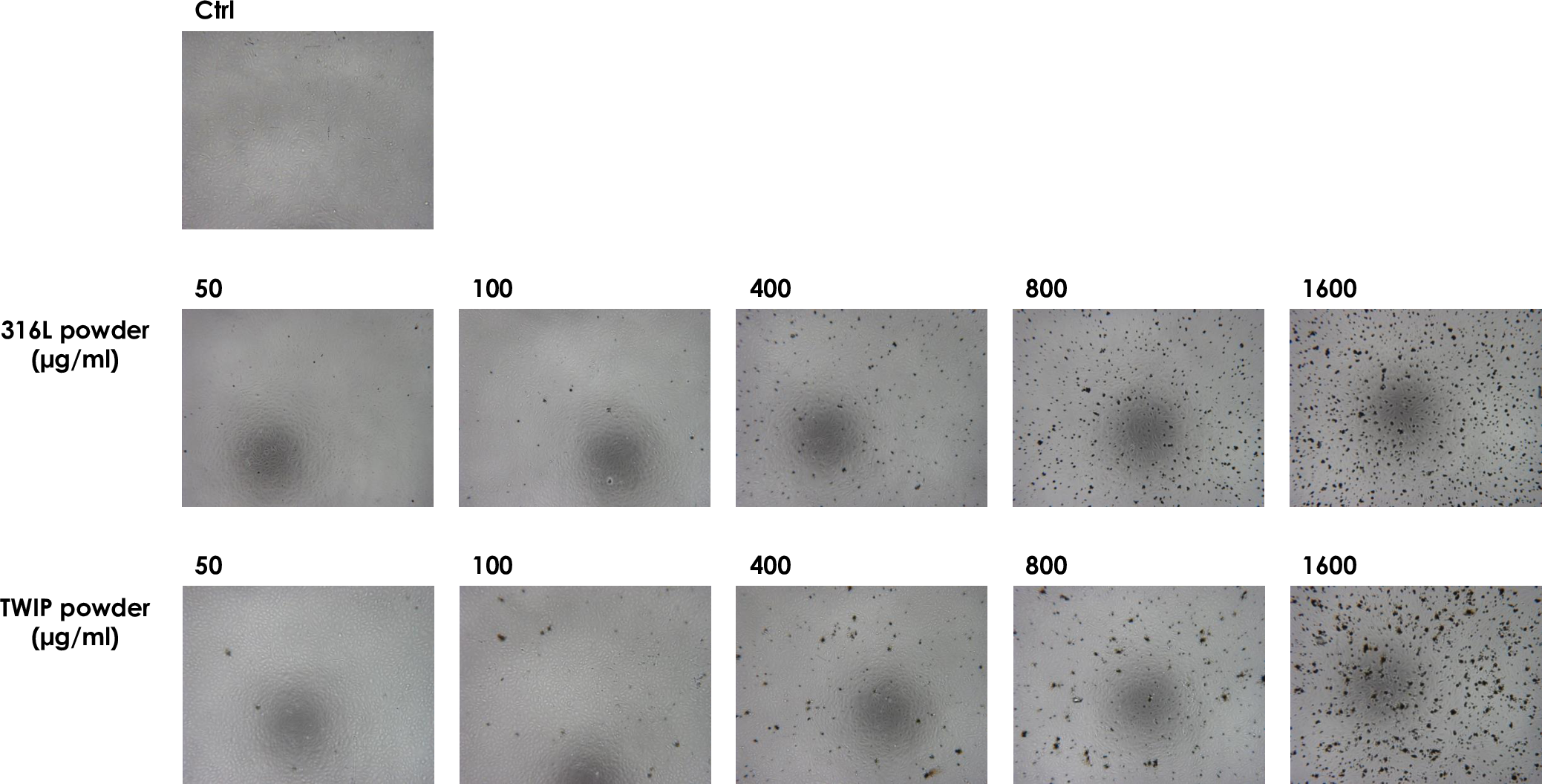

Supplement: S2 Fig — HUVECs (20,000 cells/well) were seeded into 96-well plates and exposed the day after to increasing concentration of 316L steel or TWIP steel powders. A 40x magnification image of cells was taken through an optical ZEISS Axiocam microscope camera after 24h of exposure. (TIF) [file pone.0231634.s002.tif]

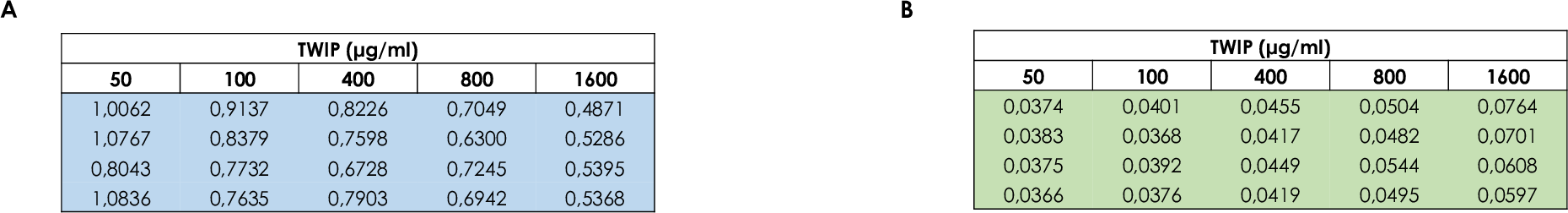

Supplement: S3 Fig — Absorbance values of TWIP steel powder at 450 nm (A) and 690 nm (B). HUVECs were cultured on 96-well plates for 24h and then exposed to increasing concentration of TWIP steel powder. After 24h, cells were washed twice with DPBS and incubated in fresh medium with 10% WST-1 reagent for 2h. Absorbance was measured at 450 nm and 690 nm in a multiplate reader (Infinite F200, Tecan®). (TIF) [file pone.0231634.s003.tif]
